# Haploinsufficiency of *ABL1* is associated with dominant isolated omphalocele

**DOI:** 10.3389/fcell.2025.1630894

**Published:** 2025-08-06

**Authors:** Caroline M. Kolvenbach, Öznur Yilmaz, Filipa M. Lopes, Jeshurun C. Kalanithy, Katharina Lemberg, Vineeta Sharma, Amar J. Majmundar, Matthias Geyer, Adrian S. Woolf, Friedhelm Hildebrandt, Benjamin Odermatt, Heiko Reutter

**Affiliations:** ^1^Department of Pediatrics, Boston Children’s Hospital, Harvard Medical School, Boston, MA, United States; ^2^Medical Faculty, Institute of Anatomy and Cell Biology, University of Bonn, Bonn, Germany; ^3^Department of Pediatrics I, University Children’s Hospital Heidelberg, Medical Faculty, Heidelberg University, Heidelberg, Germany; ^4^Medical Faculty, Institute of Neuroanatomy, University of Bonn, Bonn, Germany; ^5^Division of Cell Matrix Biology and Regenerative Medicine, School of Biological Sciences, Faculty of Biology Medicine and Health, The University of Manchester, MA, United Kingdom; ^6^Department of Neonatology and Pediatric Intensive Care Medicine, University Children’s Hospital Bonn, Bonn, Germany; ^7^Department II of Internal Medicine and Center for Molecular Medicine Cologne, University of Cologne and University Hospital Cologne, Cologne, Germany; ^8^Medical Faculty, Institute of Structural Biology, University of Bonn, Bonn, Germany; ^9^Division of Neonatology and Pediatric Intensive Care, Department of Pediatrics and Adolescent Medicine, Friedrich-Alexander University of Erlangen-Nürnberg, Erlangen, Germany

**Keywords:** omphalocele, ABL1, dominant, exome sequencing, haploinsufficiency

## Abstract

Omphalocele is a rare birth defect of the abdominal wall that results in herniation of the visceral organs through the umbilicus. To date, there are no identified genetic causes for non-syndromic isolated omphalocele. Exome sequencing in a four-generation multiplex family with isolated dominant omphalocele revealed a novel extended splice site variant (c.310 + 3A>C; p.?) in *ABL1* that encoded a non-receptor tyrosine kinase. Consistent with *in silico* predictions, in peripheral blood, this variant leads to an alternatively spliced mRNA harboring a premature termination codon. Quantification of the *ABL1* mRNA abundance showed its significant reduction in an affected allele carrier compared to a healthy control. These data indicate degradation of the aberrantly spliced transcript by non-sense-mediated decay (NMD), consistent with haploinsufficiency as the disease mechanism. Accordingly, exposure to different tyrosine kinase inhibitors during pregnancy is associated with a significantly higher risk of omphalocele in the exposed offspring. *ABL1* is a causative gene for congenital heart defect and skeletal malformation syndrome (CHDSKM) and human ABL1 deficiency syndrome (HADS); CHDSKM is associated with gain-of-function while HADS is associated with 3′ truncating variants, likely escaping NMD. Therefore, allele-dependent mechanisms may explain the phenotypic diversity. In human embryos (45–47 days post fertilization), ABL1 was immunodetected in fibroblast-like cells in the umbilical cord as well as abdominal wall surface ectoderm, both of which are important sites for abdominal wall closure. In mouse embryos (embryonic days 14.5–15.5)*,* wholemount *in situ* hybridization confirmed *Abl1* expression in the umbilical cord. Our genetic and experimental findings provide evidence that *ABL1* haploinsufficiency is the first monogenic cause for isolated dominant omphalocele.

## Introduction

Omphalocele is a rare birth defect of the abdominal wall that has a reported prevalence of 1 in 4,000 live births (Corey et al., 2014). Along with impaired regression of the physiological midgut herniation occurring normally at 11–12 weeks of gestation, the viscera remain in the dilated umbilical cord and are covered by the amnion and peritoneum to create an omphalocele (Duhamel, 1963; Khan et al., 2019; Khan et al., 2022). The prognosis of omphalocele depends on the defect size, birth weight, and severity of concomitant congenital malformations. The overall survival rate is approximately 80%, and the survival rate is over 90% for the isolated omphalocele (Conner et al., 2018; Tassin et al., 2013). To date, very little is known about the embryological pathogenesis of an omphalocele. One hypothesis states that incorrect cell migration or proliferation at the ventral body wall is a critical factor (Khan et al., 2019; Khan et al., 2022). Other studies suggest key roles of aberrant epithelial–mesenchymal interactions and essential communication between the ectoderm and mesoderm of the ventral body wall (Brewer and Williams, 2004; Mu et al., 1991). However, the exact mechanism leading to omphalocele development remains elusive. Omphalocele is frequently observed in syndromes such as trisomies (Hsu and Hou, 2007; Karaman et al., 2015; Chen, 2007) and the Beckwith–Wiedemann syndrome (Barisic et al., 2018) but may also occur in isolation. Isolated omphaloceles have been reported in 3%–10% of all prenatally diagnosed cases of the condition (Brantberg et al., 2005; Cubo et al., 2020). Most isolated cases are known to be sporadic but there are also reports on rare dominant familial occurrences (Osuna and Lindham, 1976; Frolov et al., 2010; Byron-Scott et al., 1998). The genetic basis for isolated dominantly inherited omphalocele remains unknown. Herein, we present genetic and experimental evidence indicating *ABL1* haploinsufficiency as the first monogenic cause of isolated dominant omphalocele.

## Methods

### Clinical data and specimen collection

The study was conducted in adherence to all the guidelines of the Declaration of Helsinki. Informed consent was obtained from the affected individuals or proxies in the case of minors. The study was approved by the ethics committee of the medical faculty of the University of Erlangen (no. 22-23-Bn) as well as the respective ethics committees of the collaborating centers in Boston and Manchester. Human embryonic tissues were collected after maternal consent and ethical approval (18/NE/0290 and 23/NE/0135) and were provided by the Medical Research Council and Wellcome Trust Human Developmental Biology Resource (http://www.hdbr.org/).

### Sequencing, genomic analysis, and mRNA quantification

Exome sequencing was performed as described previously (Kolvenbach et al., 2019). Here, the splice site variants were evaluated using the *in silico* prediction tools MaxEnt (http://hollywood.mit.edu/burgelab/maxent/Xmaxentscan_scoreseq_acc.html), NNSPLICE (https://www.fruitfly.org/seq_tools/splice.html), and SSF (http://www.umd.be/searchsplicesite.html) provided by AlaMut. The presence or absence of the identified variant was confirmed via Sanger sequencing for all individuals whose DNA samples were available. The total RNA samples were extracted from human lymphoblast cells from the proband III-401 and control using the PAXgene Blood RNA Kit IVD (Qiagen) and were reverse-transcribed to cDNA using the iScript cDNA Synthesis Kit (Biorad). The cDNA from each sample was then amplified by reverse-transcription polymerase chain reaction (RT-PCR) and Sanger sequenced after gel extraction. Umbilical cord tissue samples were collected immediately after birth from healthy donors at the University of Erlangen-Nuremberg and snap-frozen and the RNA were subsequently extracted using a TRIzol^TM^-based approach. Briefly, 100 mg of the tissue was homogenized in 1 mL of TRIzol^TM^ using a TissueRuptor® II. Following cell lysis with chloroform and phase separation by centrifugation, the aqueous phase containing the RNA was carefully collected. The RNA was then precipitated by reverse transcription and PCR before performing Sanger sequencing. For quantitative RT-PCR, the iTaq^TM^ Universal SYBR® Green Supermix (Biorad) was used according to manufacturer instructions. Each condition was analyzed across three experiments along with technical triplicates or duplicates per experiment. The data were normalized to the housekeeping gene *EEF1A1* belonging to the EEF gene group and expressed as mean ± standard error of the mean (SEM) (Gupta et al., 2023a, 2023b; Nazet et al., 2019; Gupta et al., 2022). Statistical significance was evaluated using the unpaired two-tailed Student’s t-test, where *p*-values less than 0.05 were considered significant. The primers used are listed in Supplementary Table S1.

### Expression analysis

Two phenotypically normal human male embryos at Carnegie Stage 19 (45–47 days post fertilization) were examined; these samples originated from elective terminations. The paraffin sections were cut and processed for immunostaining as described previously (Beaman et al., 2022). Briefly, after removing the paraffin and rehydrating the sections, the endogenous peroxidase activity was attenuated using hydrogen peroxide. The sections were then heated in a microwave for 10 min and cooled in an antigen retrieval solution (10 mM of sodium citrate, pH 6.0) for 20 min at room temperature. Then, overnight incubation with the primary anti-ABL1 antibody (1:200, LS-B2776, LifeSpan Biosciences) was performed at 4°C. After several washing steps, a biotinylated secondary antibody was added. The substance 3,3-diaminobenzidine (DAB) is a substrate of the peroxidase enzyme that allows detection of positive staining. The sections stained for ABL1 were additionally counterstained with hematoxylin to better differentiate the cell structures. Images were then acquired using a 3D-Histech Panoramic-250 microscope slide scanner with a 20×/0.08 Plan Apochromat objective (Zeiss) and CaseViewer software (3D-Histech).

Antisense and sense probes were designed for two regions of murine *Abl1*. One of the probes targeted the 5′UTR (Primer-Abl1-5′UTR-F: CATCATAAGCTTCTCCACACTCCCTGCTTCTC; Primer-Abl1-5′UTR-R: CATCATGGATCCTGCTTAGCCGCTCCTACTTC) region that is less conserved among paralogs, thereby increasing the specificity of the probe. The second probe targeted a region of the *Abl1* cDNA (Primer-Abl1-e4e5-F [spanning exons]: CATCATAAGCTTCCGTGAAGACCTTGAAGGAG; Primer-Abl1-e9-R: CATCATGGATC CATGGTTTCAAAGGCTTGGTG). At the 5′ ends of the primers, we added recognition sites for the restriction enzymes *BamHI* and *HindIII* along with a short arbitrary sequence (CATCAT) to facilitate enzyme binding. The *Abl1* probes were amplified from murine cDNA and cloned in pBluescript. The constructs were linearized with corresponding restriction enzymes, and Dig-labeled RNA was synthesized using the Roche Dig labeling kit. Mouse embryos (embryonic days 14.5–15.5) were fixed overnight in 4% paraformaldehyde (PFA) at 4°C. The endogenous peroxidase activity was subsequently quenched by incubation in 30% hydrogen peroxide on ice for 1 h. Permeabilization was carried out using proteinase K (5 μg/mL) in calcium- and magnesium-free Dulbecco’s phosphate-buffered saline (PBS) containing 0.1% Tween® 20 (DPBST) for 1 h, followed by a second fixation in 4% PFA and 0.2% glutaraldehyde buffer for 20 min at room temperature. The embryos were then incubated in a hybridization buffer (50% formamide, 5× saline-sodium citrate, 1% sodium dodecyl sulfate (SDS), 50 μg/mL of heparin, and 50 μg/mL of Torula yeast RNA IV) for 1 h at 70°C. The generated *in situ* probes were added at a final concentration of 0.5 μg/mL to the hybridization buffer and incubated overnight at 70°C. On the following day, after several washes with PBST, the samples were treated with RNase to reduce non-specific RNA hybridization. After blocking with TBST containing 10% heat-inactivated sheep serum at room temperature, the embryos were incubated overnight at 4°C with an alkaline-phosphatase-conjugated anti-fragment antigen-binding (Fab) antibody (1:5,000; Roche-11093274910, Roche). On the third day, the samples were washed in alkaline phosphatase buffer and stained using BM Purple AP substrate until the desired signal intensity was achieved. The staining reaction was stopped with 5 mM of EDTA in PBST. The fully stained embryos were then stored in 100% glycerol and imaged using a Leica M205C system equipped with a color camera. Both generated antisense probes showed matching staining.

## Results

**FIGURE 1:**
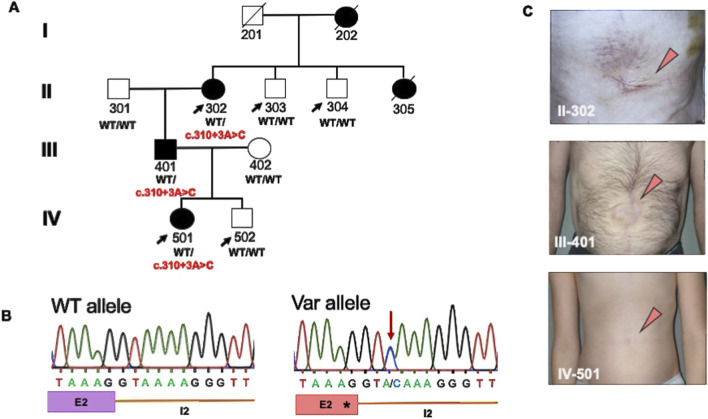
Identification of a heterozygous extended splice site variant in *ABL1* in a multiplex family with omphalocele. **(A)** Pedigree of the multiplex family showing compatibility with an autosomal dominant mode of inheritance. The individuals subjected to exome sequencing are highlighted with an arrow. DNA samples were available for eight family members, as outlined in the pedigree. The affected individuals II-302, III-401, and VI-501 carried a novel extended splice site variant (c.310 + 3A>C) in *ABL1*, which was absent in all tested healthy family members. Individuals II-302 and IV-501 presented with isolated omphaloceles, whereas III-401 additionally had inguinal and diaphragmatic hernia. **(B)** Chromatograms of the heterozygous variant (Var) and wildtype (WT) allele detected in ABL1 residing in the extended splice donor site of intron (I) 2. **(C)** Postoperative ventral images after neonatal abdominal wall closures of II-302, III-401, and IV-501.

Herein, we describe a previously unreported multiplex family (Figure 1) of five individuals with isolated omphalocele occurring over four generations, consistent with autosomal dominant mode of inheritance. Exome sequencing was performed for the unaffected (II-303, II-304, and IV-502) and affected (II-302 and IV-501) family members, and a novel extended splice site variant was identified in *ABL1* (c.310 + 3A>C; p.?) (Figure 1A). Sanger sequencing was used to confirm the presence of the variant in all affected family members whose DNA samples were available (II-302, III-401, and IV-501; Figures 1A,B) as well as the absence of the variant in all clinically unaffected individuals (II-301, II-303, II-304, III-402, and IV-502; Figures 1A,B).

**FIGURE 2:**
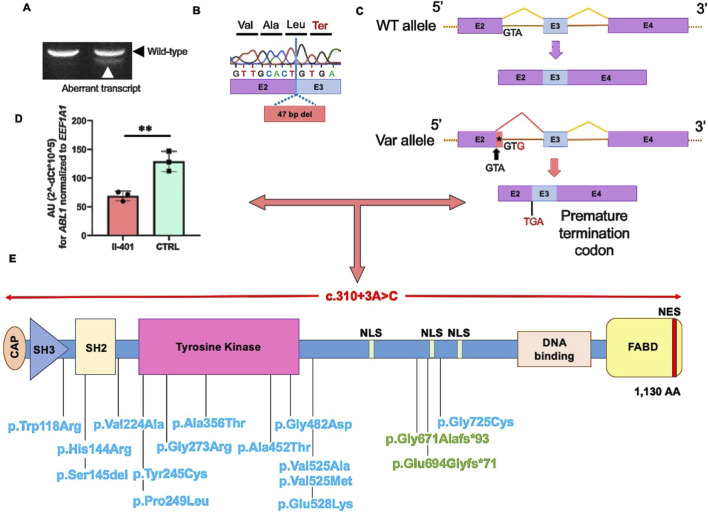
Identified splice variant led to alternative splicing and reduction of *ABL1* mRNA expression in the affected proband. **(A)** Gel electrophoresis of the reverse-transcribed and amplified cDNA revealed the presence of an additional alternatively spliced shorter PCR product (white arrowhead, aberrant transcript) compared to the healthy control (black arrowhead, wild type). **(B)** Sanger sequencing of the amplified additional alternatively spliced shorter cDNA product demonstrated the loss of 47 bp of exon (E) 2. **(C)** Owing to the use of an alternative splice donor (sequence GTA) within exon 2, the resulting frameshift on cDNA level leads to a premature termination codon shown here in the isoform ABL1-201 (ABL1-202 not shown). **(D)** Quantification of the ABL1 mRNA abundance *via* quantitative (q) RT-PCR showed a significant reduction in III-401 (red) compared to a healthy control (green, CTRL). These data indicate the degradation of the aberrantly spliced transcript by non-sense-mediated decay (NMD). AU: arbitrary units; **p < 0.01 by Student’s t-test. **(E)** Schematic representation of the ABL1 protein with the identified heterozygous loss-of-function (red, omphalocele), reported biallelic truncating (green, human ABL1 deficiency syndrome (HADS)), and heterozygous gain-of-function (blue, congenital heart defects and skeletal malformations syndrome (CHDSKM)) variants. Source: The Human Gene Mutation Database (HGMD Professional v2024.4) and literature (Wang et al., 2017; Chen et al., 2020; Blakes et al., 2021; AlAbdi et al., 2024). FABD, F-actin-binding domain; NES, nuclear export signal; NLS, nuclear localization signal; SH3, Src homology 3 domain; SH2, Src homology 2 domain.

The splice variant resides in the splice donor site of intron 2 of *ABL1*, affecting the canonical *ABL1*-201 (ENST00000318560.6) and non-canonical *ABL1*-202 (ENST00000372348.9) transcripts that differ by an alternatively spliced first exon. The predicted change using the *in silico* splicing tools at the donor site is 53% (MaxEnt: 78%; NNSPLICE: 69%; SSF: 12%). Based on this prediction, we sought the potential effects on splicing in isolated peripheral blood RNA from individual III-401 (Figures 1, 2). Gel electrophoresis of the reverse-transcribed and amplified cDNA for *ABL1* revealed the presence of an additional alternatively spliced product compared to the healthy control in both transcripts (Figure 2A; *ABL1*-202 not shown). Sanger sequencing of the amplified cDNA product demonstrated loss of 47 bp of exon 2 owing to an alternative early splice donor in intron 2 (Figure 2B). The resulting frameshift led to the incorporation of a premature termination codon (PTC) in both transcripts (Figures 2B,C). PTCs located more than 50–55 nucleotides upstream of the last exon–exon junction typically trigger non-sense-mediated decay (NMD), whereas those closer to the 3′ end often escape NMD, resulting in truncated but stable proteins (Supplementary Figure S1) (Gupta et al., 2023b). Quantitative determination of the *ABL1* mRNA abundance showed a significant (*ABL1-201*) or partial (*ABL1-202*) reduction in III-401 compared to a healthy control (Figure 2D; Supplementary Figure S2). These data confirm the degradation of the aberrantly spliced transcript by NMD and loss-of-function with haploinsufficiency reducing the overall gene dosage as the underlying disease pathomechanism. The more modest and statistically non-significant reduction observed for transcript *ABL1-202* may reflect isoform-specific differences in the splicing efficiency, expression levels, or susceptibility to NMD (Supplementary Figure S2B). This analysis serves as a preliminary functional support for a potential transcriptional effect of the variant despite the low sample number.

**FIGURE 3:**
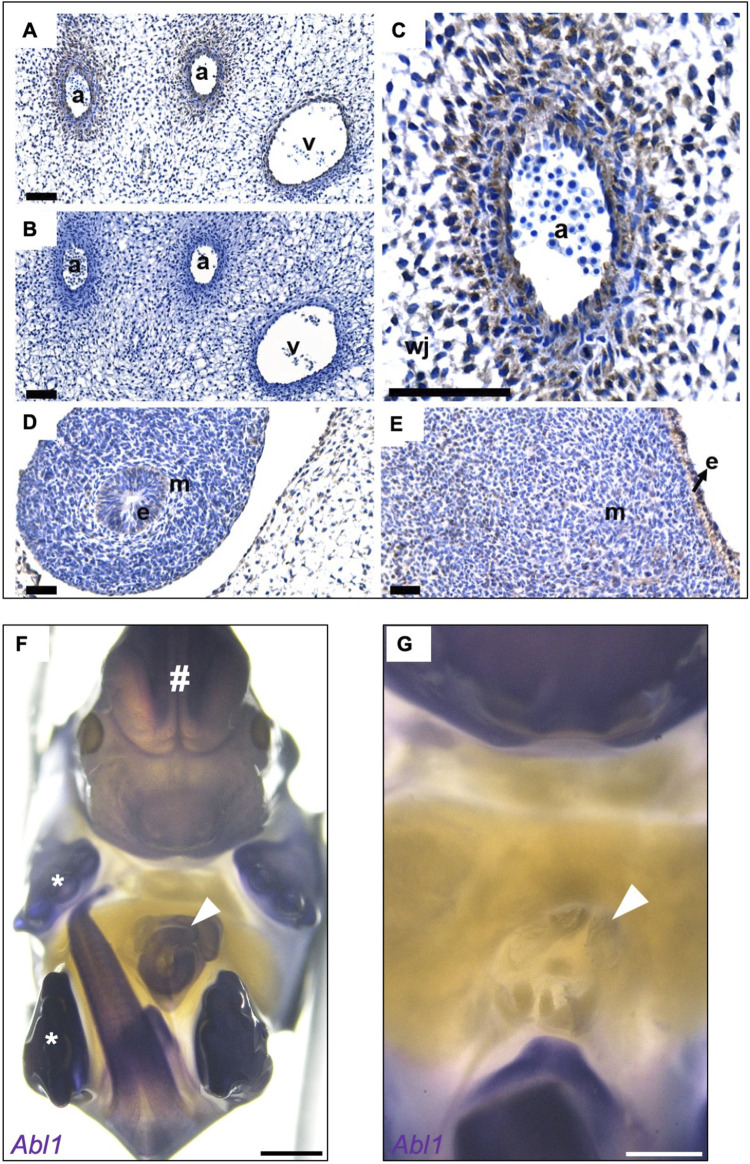
ABL1 is expressed in the abdominal walls and umbilical cords of human and mouse embryos. **(A)** Human embryonic immunohistochemistry for ABL1 for representative sections from two Carnegie Stage 19 embryos. The positive immunostaining for ABL1 is shown in brown color, while the nuclei are counterstained blue with hematoxylin. Immunostaining of the umbilical cord in the arteries (a) and vein (v). **(B)** No brown signal was detected in the negative control, where the primary antibody was omitted. **(C)** Prominent ABL1 signals in the wall of the umbilical artery (a) and in adjacent fibroblast-like cells extending into Wharton’s jelly (wj). **(D)** ABL1 signal was detected in the epithelium (e) of the physiologically herniated small intestine; the surrounding mesenchyme is indicted by m. **(E)** Epidermis (e) on the ventral surface of the trunk was positive for ABL1 along with a subset of cells in the underlying mesenchyme (m). **(F)** Wholemount *in situ* hybridization in mouse embryos on embryonic day 14.5 show strong staining of *Abl1* in the umbilical cord (white arrowhead). Furthermore, positive staining was observed in the tissues from the brain (#), limbs (asterisks), outer genitals, and tail. **(G)** Magnification of the image in F after removal of the umbilical cord, umbilical stem, or umbilical ring; the surrounding abdominal wall tissues do not show expression of *Abl1*. Dpf, days post fertilization. Scale bars: A–E (40 μm); F and G (1,000 µm).

Next, we sought the embryonic expression of ABL1 in the region of the nascent umbilicus, which is the site where an omphalocele is formed. Two phenotypically normal human embryos at Carnegie Stage 19 (45–47 days post fertilization) with similar immunostaining patterns for ABL1 were examined (Figure 3A). At this point in time, organogenesis of diverse viscera is ongoing, and the small gut is herniated through the anterior wall of the embryo (Bouzada et al., 2022). Prominent immunostaining was detected within the umbilical cord (Figure 3A). No signal was detected in the negative control where the primary antibody had been omitted (Figure 3B). Specifically, ABL1 was immunodetected in the walls of the umbilical arteries, and several layers of the surrounding fibroblast-like cells were also positive (Figure 3C). A weak ABL1 signal was detected in the epithelium of the small intestine (Figure 3D) situated outside the embryo after being physiologically herniated. The skin on the ventral surface of the embryonic trunk was also immunostained for ABL1, with notable signals in both the epidermis and in a subset of the underlying mesenchymal-like cells (Figure 3E). The presence of different *ABL1* mRNAs (*ABL1*-201 and *ABL1*-202) in the umbilical cords of human newborns was confirmed by RT-PCR and subsequent Sanger sequencing (Supplementary Figure S3). We also conducted *in situ* hybridization (ISH) in healthy wild-type (WT) mouse embryos that showed strong *Abl1* expressions in the umbilical cord on embryonic day (E) 14.5 (Figure 3F) shortly before abdominal wall closure on E15.5 (Nichol et al., 2011). *Abl1* expressions were also noted in the embryonic brain, limbs, tail, and forming external genitalia (Figure 3F). Conversely, *Abl1* expressions were not detected in the abdominal wall structures around the umbilicus (Figure 3G).

## Discussion

ABL1 encodes a phosphotyrosine kinase that is widely expressed and implicated in cell adhesion, division, and differentiation (Wang, 2014). The gene is well-recognized in the pathogenesis of chronic myeloid leukemia (CML), where translocation of chromosomes 22 and 9 creates the BCR-ABL1 fusion gene (Quintás-Cardama and Cortes, 2006). Heterozygous gain-of-function missense mutations in *ABL1* have been reported to cause congenital heart defect and skeletal malformation syndrome (CHDSKM; Figure 2E, blue variants) (Wang et al., 2017; Chen et al., 2020; Blakes et al., 2021). Although CHDSKM does not feature the classic omphalocele, some individuals have umbilical or diaphragmatic hernias or abdominal musculature hypotonia. Moreover, functional dysmotility and intestinal malrotation have been reported.

Recently, AlAbdi et al. (2024) described two multiplex consanguineous families, where each carried biallelic truncating variants in *ABL1* that caused human ABL1 deficiency syndrome (HADS; Figure 2E, green variants). The affected individuals presented with multiple congenital malformations and distinct facial dysmorphology, such as a short, depressed, and broad nose, long philtrum, and macrostomia (AlAbdi et al., 2024). These characteristics were not present in the affected individuals of the multiplex family described herein. The identified variants were located in the last exon of *ABL1*, probably leading to NMD escape. Protein-truncating variants in the final coding exon are anticipated to escape NMD; this hypothesis is supported by the *Abl1* knockout mouse model, where a neomycin resistance cassette was inserted downstream of the tyrosine kinase domain (AlAbdi et al., 2024; Schwartzberg et al., 1989). The resulting shortened protein contained a functional kinase domain but lacked the DNA and actin binding domains (Figure 2E) (AlAbdi et al., 2024; Schwartzberg et al., 1989). If the 3′ truncating variants escape NMD and code for short mutant proteins with preservation of the tyrosine kinase domain, these proteins may be functional with attenuated or altered natures. Consequently, allelic diversity may explain the observed phenotypic differences between syndromic CHDSKM or HADS and the isolated omphalocele.

Interestingly, omphaloceles have been repeatedly reported in newborns with gestational exposure to tyrosine kinase inhibitors (Chelysheva et al., 2018; Abruzzese et al., 2022; Étienne et al., 2010; Madabhavi et al., 2019; Pye et al., 2008). These observations are summarized in Supplementary Table S2. For example, omphaloceles occurred in three out of 125 pregnancies exposed to imatinib, which is one such drug (Supplementary Table S2) (Pye et al., 2008). Comparable observations have been reported for exposures to nilotinib and dasatinib during pregnancy. Since the expected birth prevalence of omphaloceles in the normal population is approximately 1 in 4,000 births, embryonic or fetal exposure to imatinib would increase the risk by 100-fold. Potential confounding factors like the severity of the underlying maternal disease and co-medications may also contribute to the observed outcomes. The fact that exposure to different tyrosine kinase inhibitors along with downregulation of ABL1 activity during pregnancy is associated with a significantly higher risk of omphalocele in the exposed offspring supports our hypothesis that the genetically induced *ABL1* haploinsufficiency observed herein led to omphaloceles in the reported variant carriers.

Omphalocele is a midline defect at the amnio-ectodermal transition zone at the umbilical ring. The importance of epithelial–mesenchymal interactions has been demonstrated with the example *Tfap2a* (*AP-2α)* mouse model (Brewer and Williams, 2004). Conditional knockout of *Tfap2a* leads to abdominal wall defects, and *AP-2α:LacZ* studies have demonstrated expressions in the surface ectoderm of the ventral and dorsal body walls at E15.5 (Brewer and Williams, 2004). In our study, ABL1 was detected in the surface ectoderm and underlying mesenchymal structures. Loss of ABL1 expressions in the ventral ectoderm and fibroblasts could disrupt key cellular processes such as adhesion, migration, and cytoskeletal organization, all of which are critical for ventral body wall closure. These functions overlap with those of AP-2α. ABL1 could act in coordination with or downstream of the AP-2α-regulated pathways, suggesting a potential role of ABL1 in epithelial–mesenchymal interactions. The absence of a detectable *Abl1* signal in the mouse abdominal wall upon ISH could reflect the lower sensitivity compared to human immunostaining. Additionally, species-specific differences in gene regulation or developmental timing could contribute to the observed variations in expression patterns. Collectively, our expression data for ABL1 in human and mouse developments are consistent with the important roles of ABL1 in physiological gut herniation and body wall closure owing to impacts on the biologies of both the umbilical cord and nearby anterior body wall.

In summary, we identified an extended splice variant in *ABL1* leading to haploinsufficiency in a multiplex family with isolated dominant omphalocele. Quantification of the *ABL1* mRNA abundance showed its significant reduction in an affected allele carrier compared to a healthy control, indicating degradation of the aberrantly spliced transcript by NMD. The phenotypic consistency, *ABL1* mRNA quantification, and variant prediction in the context of NMD suggest haploinsufficiency as the underlying disease mechanism, underscoring the need for future protein-level studies. Exposure to different tyrosine kinase inhibitors during pregnancy is associated with omphalocele in the exposed offspring. Expression studies in healthy human and mouse embryo bodies show expressions of ABL1 in the relevant tissue structures for body wall closure and the umbilical cord. Identification of *ABL1* variants in families with a history of omphalocele could provide valuable insights into the recurrence risk and enable genetic counseling. Moreover, given the observed association between *ABL1* and omphalocele, the findings may warrant increased clinical awareness or consideration of genetic screening in pregnancies with known exposures to tyrosine kinase inhibitors, although further studies are required to establish a direct causal relationship. Based on these genetic and experimental findings, we have presented the first description of a monogenic cause for isolated dominant omphalocele.

## Data Availability

The datasets generated during this study are available from the corresponding authors upon request.
