# Supplementary figures and images for: Haploinsufficiency of ABL1 is associated with dominant isolated omphalocele
Source: Front Cell Dev Biol. 2025 Aug 6;13:1630894. doi: 10.3389/fcell.2025.1630894 (PMC12365408; doi:10.3389/fcell.2025.1630894)

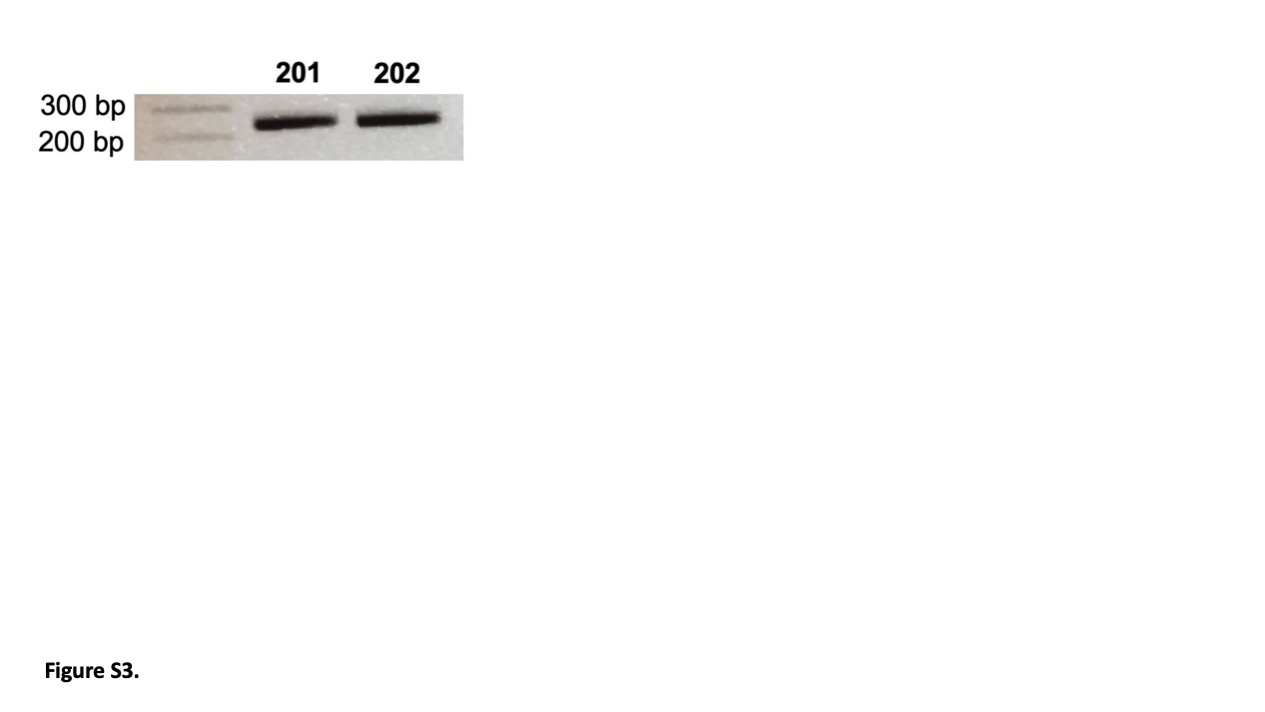

Supplement: Supplementary file 1 [file Image3.jpeg]

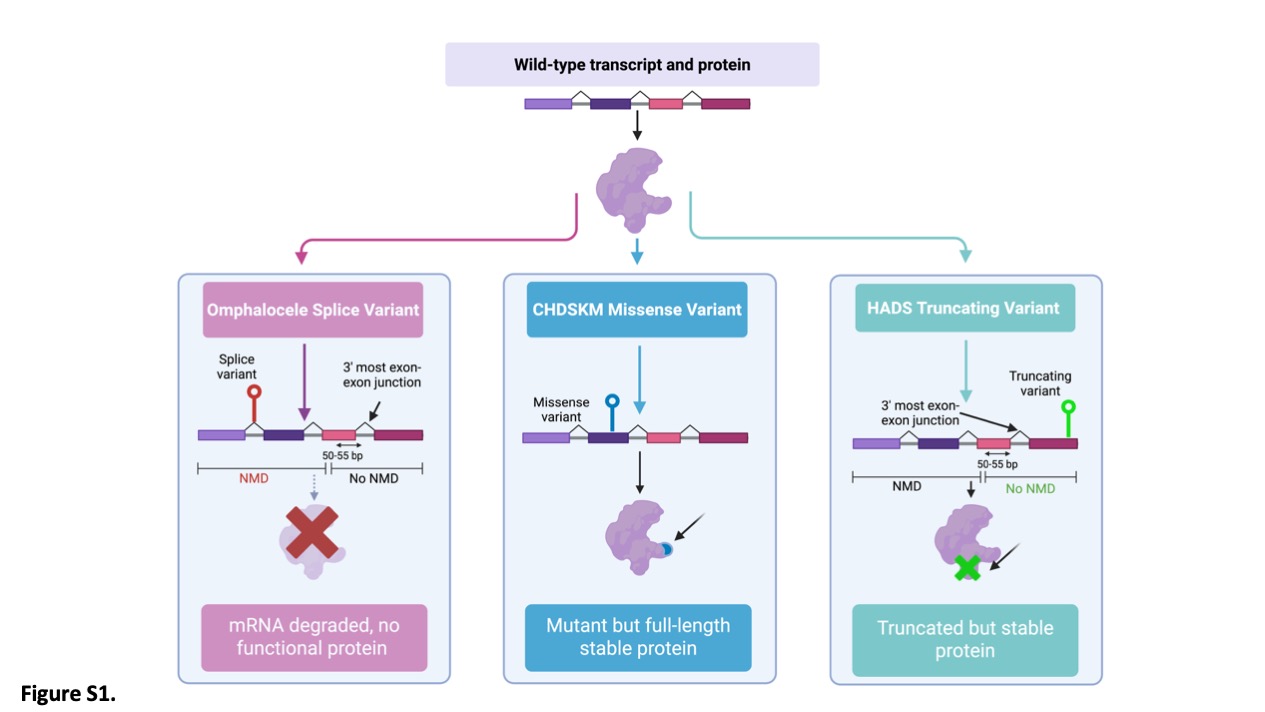

Supplement: Supplementary file 2 [file Image1.jpeg]

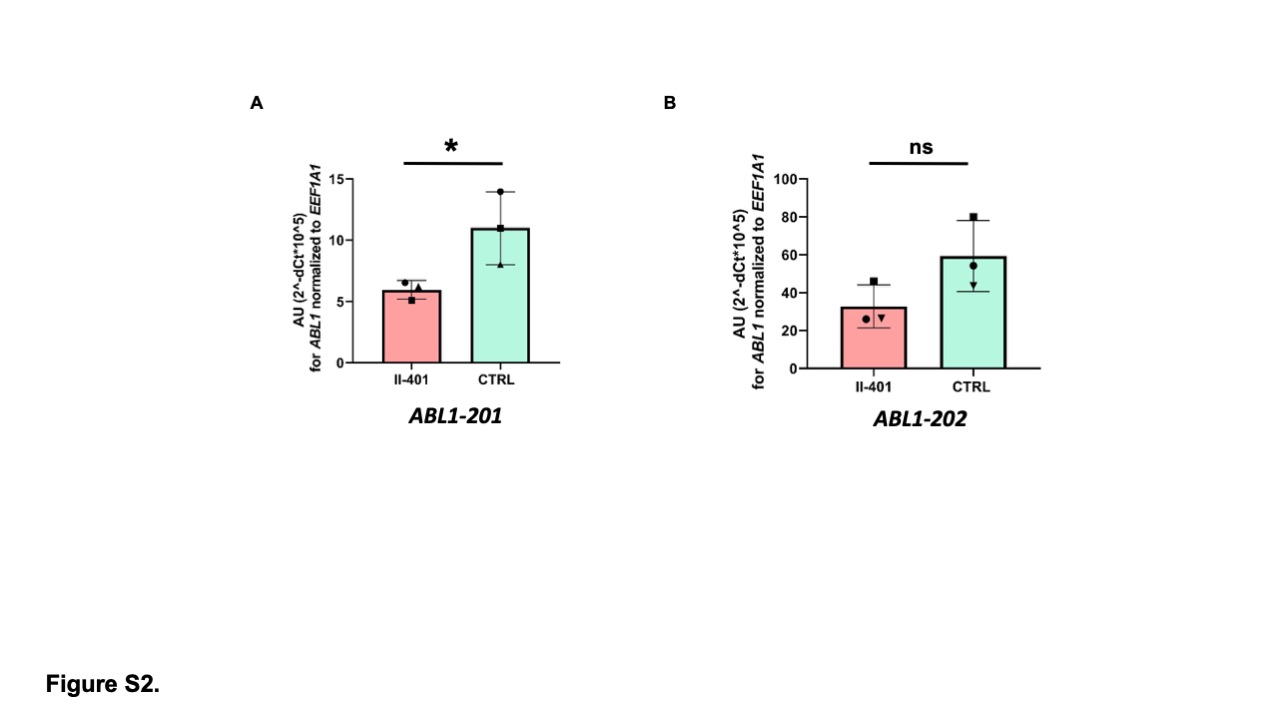

Supplement: Supplementary file 3 [file Image2.jpeg]
