# Supplementary material for: Haploinsufficiency of ABL1 is associated with dominant isolated omphalocele
Source: Front Cell Dev Biol. 2025 Aug 6;13:1630894. doi: 10.3389/fcell.2025.1630894 (PMC12365408; doi:10.3389/fcell.2025.1630894)
Supplement: Supplementary file 4 [file DataSheet1.docx]

**SUPPLEMENTAL FIGURES AND TABLES**

**FIGURE LEGENDS**

**Figure S1. Schematic illustration of the predicted genetic outcomes of different variant types in *ABL1*.**

Splice variants typically lead to premature termination codons (PTCs) more than 50–55 base pairs (bp) upstream of the most 3’ exon–exon junction, causing nonsense-mediated decay (NMD) and resulting in no protein (left panel). Congenital heart defects and skeletal malformations (CHDSKM) syndrome missense variants result in full-length proteins with potentially altered function (middle panel). In contrast, truncating variants in the last exon downstream of the last exon-exon junction in human ABL1 deficiency syndrome (HADS) possibly escape NMD, producing truncated proteins (right panel). Adapted from Maquat et al.(1). Created in BioRender. Saida, K. (2025) https://BioRender.com/dqs39rh.

**Figure S2. Identified splice variant leads to a reduction of *ABL1* mRNA expression in affected proband.**

**A-B)** Quantification of the *ABL1* mRNA abundance via qRT-PCR showed a significant (**A;** *ABL1*-201, ENST00000318560.6) or partial (**B;** *ABL1*-202, ENST00000372348.9) reduction of *ABL1* mRNA in III-401 (red) compared to a healthy control (green, CTRL). These data indicate the degradation of the aberrantly spliced transcript by NMD. AU: arbitrary units; *, p<0.05 student’s t-test

**Figure S3. mRNA expression of *ABL1* in human newborn umbilical cord tissue.**

Using RT-PCR, the expression of both *ABL1* mRNA transcripts (*ABL1-201*, ENST00000318560.6 and *ABL1-202*, ENST00000372348.9) could be confirmed in the umbilical cord of a healthy human newborn.

| **Primer ID** | **Sequence** |
| --- | --- |
| ABL1_201_e1e2_F | TGGAAGAAGCCCTTCAGC |
| ABL1_e3_R1 | TTGGTTTGGGCTTCACACC |
| ABL1_202_e1e2_F | GAACATGAAGCCCTTCAGC |
| EEF1A_1F | GGCATCGACAAAAGAACCAT |
| EEF1A1_1R | CCCAGGCATACTTGAAGGAG |
| EEF1A1_2F | GCTGCTGAGATGGGAAAGG |
| EEF1A1_2R | ACAGTCAGCCTGAGATGTCC |
| ABL1_202_e1_F | CTGACTTGTGGAGATGCAGC |
| ABL1_201_e1_F | TGGGCTGCAAATCCAAGAAG |
| ABL1_e3_R2 | AGGTTTTCCTTGGAGTTCC |
| ABL1_e7_F | TGAGCAGGTTGATGACAGG |
| ABL1_e8_R | CTCATACACCTGGGACAG |

**Table S1. Primers and respective sequences used for RT- and RT-qPCR.**

| **Reference** | **Trimester exposure** | **Tyrosine kinase Inhibitor** | **Fetal anomaly** | **No. omphalocele / total number of exposed embryos/fetuses** |
| --- | --- | --- | --- | --- |
| **Case series** | | | | |
| **Pye et al.**(2) | first | Imatinib | Scoliosis, **small exomphalos** | **3 / 125** |
|  | unkown | Imatinib | **Exomphalos**, right renal agenesis and hemivertebrae |  |
|  | first | Imatinib | Hypoplastic lungs, **exomphalos**, left duplex kidney, right absent kidney, hemivertebrae, and right shoulder anomaly |  |
| **Madabhavi et al.**(3) | unknown | Imatinib | **Omphalocele** | **1 / 10** |
| **Chelysheva et al.**(4) | unknown | Nilotinib | **Omphalocele** | **1 / 60** |
| **Abruezze et al.**(5) | unknown | Dasatinib | **Omphalocele** | **1 / 80** |
|  |  |  |  | **Total 6 / 275 (~ 1 / 46)** |
| **Case report** | | | | |
| **Étienne et al**.(6) | first | Nilotinib | **Large omphalocele** | **1** **/ 2** |

**Table S2. Reported pregnancies with tyrosine kinase inhibitors (TKIs) exposure to the embryo/fetus and omphalocele as pregnancy outcome** (from ancient Greek umbilicus, and hernia; also spelled omphalocele; synonym: exomphalos)

**REFERENCES**

1. Maquat LE. Nonsense-mediated mRNA decay: splicing, translation and mRNP dynamics. Nat Rev Mol Cell Biol. 2004 Feb;5(2):89–99.

2. Pye SM, Cortes J, Ault P, Hatfield A, Kantarjian H, Pilot R, et al. The effects of imatinib on pregnancy outcome. Blood. 2008 Jun 15;111(12):5505–8.

3. Madabhavi I, Sarkar M, Modi M, Kadakol N. Pregnancy Outcomes in Chronic Myeloid Leukemia: A Single Center Experience. J Glob Oncol. 2019 Sep;5:1–11.

4. Chelysheva E, Turkina A, Polushkina E, Shmakov R, Zeifman A, Aleshin S, et al. Placental transfer of tyrosine kinase inhibitors used for chronic myeloid leukemia treatment. Leuk Lymphoma. 2018 Mar;59(3):733–8.

5. Abruzzese E, Aureli S, Bondanini F, Ciccarone M, Cortis E, Di Paolo A, et al. Chronic Myeloid Leukemia and Pregnancy: When Dreams Meet Reality. State of the Art, Management and Outcome of 41 Cases, Nilotinib Placental Transfer. Journal of Clinical Medicine. 2022 Jan;11(7):1801.

6. Étienne G, Milpied B, Réa D, Rigal-Huguet F, Tulliez M, Nicolini FE. Recommandations du groupe Fi-LMC pour la gestion des effets indésirables du traitement par nilotinib (Tasigna®) au cours de la leucémie myéloïde chronique. Bulletin du Cancer. 2010 Aug 1;97(8):997–1009.
